# Tenecteplase versus Alteplase for the Management of Acute Ischemic Stroke in a Low-income Country–Nepal: Cost, Efficacy, and Safety

**DOI:** 10.7759/cureus.2178

**Published:** 2018-02-09

**Authors:** Gaurav Nepal, Ghanshyam Kharel, Shaik Tanveer Ahamad, Babin Basnet

**Affiliations:** 1 Maharajgunj Medical Campus, Tribhuvan University Institute of Medicine; 2 Department of Medicine, Deccan College of Medical Sciences

**Keywords:** alteplase, tenecteplase, nepal, low income country, ischemic stroke, ischemic stroke

## Abstract

Intravenous alteplase is the only approved treatment for acute ischemic stroke. Tenecteplase, a genetically engineered, mutant tissue plasminogen activator, is an alternative thrombolytic agent. The economic feasibility of stroke treatment has been a matter of huge debate and discussion thus far. The use of thrombolytics for the management of ischemic stroke has recently begun in Nepal. In low-income countries like Nepal, where the per capita income falls at just $691.7 and 25.2% of the population are under the poverty line, stroke patients cannot meet treatment expenses. Tenecteplase is easily available (for the management of acute coronary syndrome) in tertiary-level hospitals of Nepal and the price quote of tenecteplase ($450) is half the price of alteplase ($1000). In emergency cases, sometimes, the cost of alteplase can be greater than the patient can afford and they can't undergo thrombolysis even after arriving on time. However, evidence exists that supports the use of other alternatives (tenecteplase), which are also effective in the management of acute ischemic stroke. In this article, we examined current evidence for the efficacy and safety of tenecteplase when compared to alteplase. This review will make neurologists in Nepal familiar with the efficacy and safety of tenecteplase in comparison with alteplase since it is common for patients to not be able to afford the expensive alteplase, which makes guideline-based practice impossible some times.

## Introduction and background

Intravenous thrombolysis with alteplase remains the standard of care prior to thrombectomy for eligible patients within 4.5 hours of ischemic stroke onset [1]. Although it significantly improves the likelihood of disability-free recovery, alteplase has several limitations, such as low recanalization rate, the risk of intracranial hemorrhage (ICH), and short half-life, requiring continuous infusion [1-2]. Tenecteplase is a modified recombinant tissue plasminogen activator molecule (tPA) engineered to improve efficacy through increased affinity binding to fibrin, greater resistance to inactivation by plasminogen activator inhibitor-1, no procoagulant effects, and longer free plasma half-life [3].These pharmacodynamic differences result in more rapid coronary reperfusion, with tenecteplase now the first-line IV thrombolytic for myocardial infarction [4-5]. In animal stroke models, tenecteplase leads to more rapid and complete reperfusion than alteplase, with reduced intracranial hemorrhage [6].

The economic feasibility of stroke treatment has been a matter of huge debate and discussion thus far. The use of thrombolysis for the management of ischemic stroke has recently begun in Nepal [7]. In low-income countries like Nepal, where the per capita income falls to just $691.7 and 25.2 % of the population are under the poverty line, stroke patients cannot meet the treatment expenses [8]. Tenecteplase is easily available (for the management of acute coronary syndrome) in tertiary-level hospitals of Nepal and the price of tenecteplase ($450) is half the price of alteplase ($1000). In emergency cases, sometimes, the cost of alteplase can be greater than the patient can afford and can't undergo thrombolysis even after arriving on time. However, evidence exists that supports the use of other alternatives (tenecteplase) that are also effective in the management of an acute ischemic stroke. In this article, we examined current evidence for the efficacy and safety of tenecteplase when compared to alteplase. This review will make neurologists from Nepal familiar with the efficacy and safety of tenecteplase in comparison with alteplase since it is common for patients to not be able to afford expensive alteplase, which makes guideline-based practice impossible fewer times.

## Review

Methods

This review was conducted using standard relevant publications indexed in PubMed, PubMed Central (PMC), EMBASE, and Google Scholar. We searched using the following keywords: tenecteplase, ischemic stroke, alteplase vs. tenecteplase, thrombolysis, and cerebrovascular infarction. A thorough review of the references revealed further relevant articles. Two researchers (first and second author) individually searched and screened the attained literature. We included all relevant quantitative studies published between 2005 and 2017, published in the English language, studies that report National Institutes of Health Stroke Scale (NIHSS) at baseline and 24 hours, three-month modified ranking scale (mRS), ICH incidence, and mortality. Studies that appeared to be biased and that misconstrued data were excluded.

In total, 22 articles were shortlisted, identified, and screened but after exclusion, we selected only four articles. A data extraction form was developed in Microsoft Excel. Two researchers separately extracted data using the data extraction file, which was again cross-checked by each other. We finally analyzed the data under two broad themes: efficacy and safety. There were too many differences in the outcome measures of studies, so a quantitative analysis of data was deemed inappropriate. A qualitative summary of the data was consequently completed.

Results and discussion

Studies and Patients

**Figure 1:**
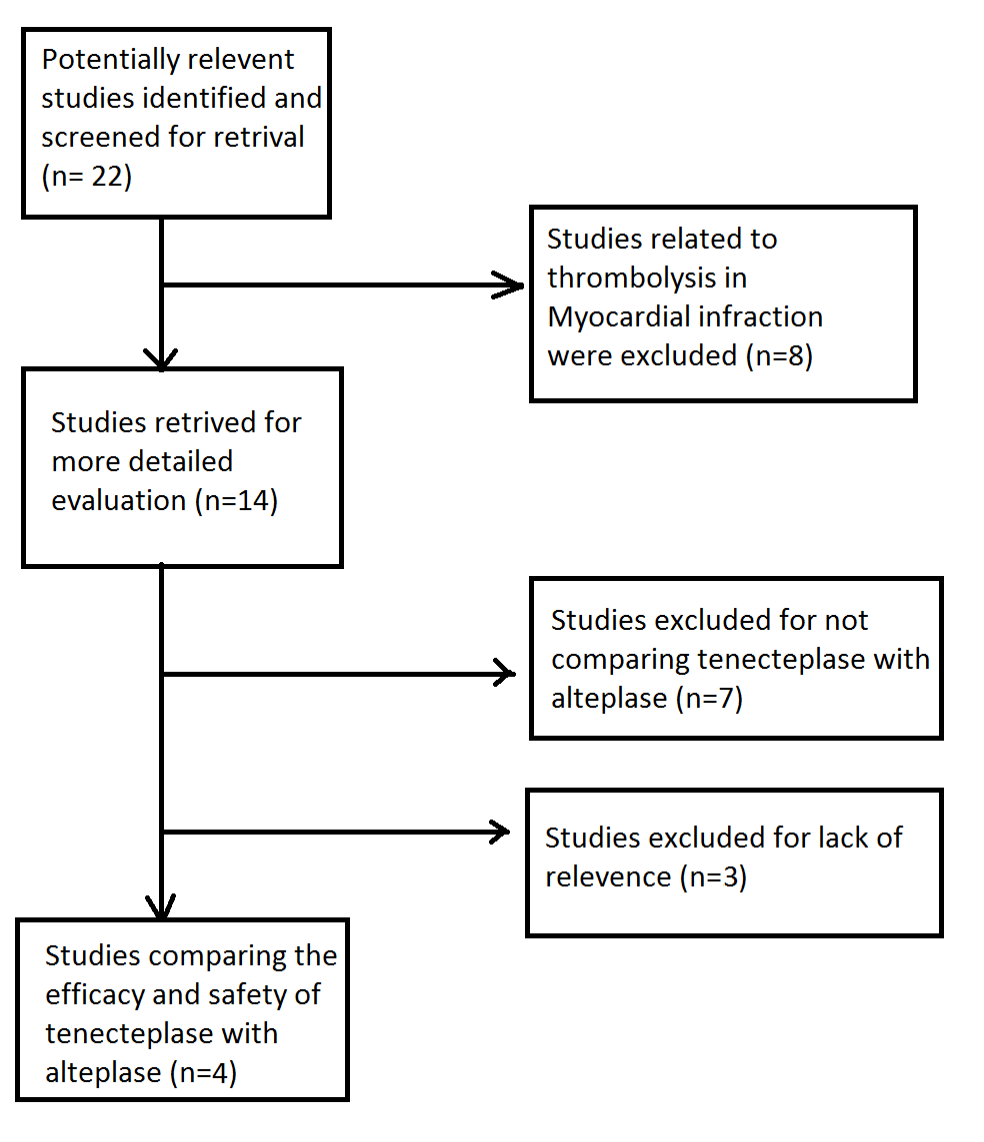
Flow diagram of included studies

All the four articles included in this review were of high quality, considering the presence of clear objectives, a clearly mentioned study design, the random sampling technique, adequate sample size, and a clearly described statistical analysis. Four trials of tenecteplase versus alteplase were included in this systematic review, with a total of 1359 patients. The overall study selection process is displayed in Figure 1. The key methodological characteristics are identified in Table 1 and further details are reported elsewhere. We concluded that the risk of bias was low across all the trials. Most trials included patients with different stroke severities and etiological subtypes although the results were not reported by subtype in most trials.

Comparison of Treatment

All studies used alteplase 0.9 mg/kg to a maximum of 90 mg (10% initial bolus, followed by 90% infused over 1 hour as the comparator). Haley et al. (Table 1) examined three different tenecteplase (TNK) doses (0.1, 0.25, and 0.4 mg/kg). The 0.4 mg/kg dose was eliminated early in the study with only 19 patients in this group because it met the prespecified criterion for the elimination of unpromising performance (a score of six fewer units than the dose group with the leading score). When the study subsequently terminated prematurely due to slow recruitment, there were 31 patients each in the 0.1, 0.25 mg/kg, and alteplase groups. Two TNK doses were tested in Parsons et al. (0.1 and 0.25 mg/kg) while Huang et al. examined only 0.25 mg/kg. Logallo et al. examined intravenous tenecteplase 0.4 mg/kg (to a maximum of 40 mg).

**Table 1:** Key methodological characteristics of selected studies RCT: Randomized controlled trial TNK: Tenecteplase ACA: Anterior cerebral artery PCA: Posterior cerebral artery MCA: Middle cerebral artery NIHSS: National institute of health stroke scale mRS: Modified ranking scale *: Five patients received study treatment but had final diagnosis of non-stroke **: Two patients received study treatment but had final diagnosis of non-stroke

Author	Year/Country	Journal	Sample size	Study design	Inclusion criteria	Doses
Haley et al. [9]	2010/USA	Stroke	31 vs 31 vs 19 vs 31	RCT	Ischemic stroke with serious measurable deficit on NIH Stroke Scale Treatment within 3 hours of stroke onset Age 18 years or older	TNK 0.1 mg/kg versus 0.25 mg/kg versus 0.4 mg/kg versus alteplase 0.9 mg/kg
Parsons MW et al. [10]	2012/Australia	New England Journal of Medicine	25 vs 25 vs 25	RCT	First stroke (not brain stem stroke); NIHSS 4; Symptoms onset <6 h; mRS 0–2; Core volume< 1/3 of MCA or 1/2 ACA/PCA territory; Perfusion volume > 120% core, and 20 ml; Occlusion of MAC/ACA/PCA	TNK 0.1 mg/kg versus 0.25 mg/kg versus alteplase 0.9 mg/kg
Huang et al. [11]	2015/UK	Lancet Neurology	52^*^ vs 51^**^	RCT	Supratentorial stroke, NIHSS 1–25; Symptoms onset <4.5 h; mRS 0–2	TNK 0.25 mg/kg versus alteplase 0.9 mg/kg
Logallo et al. [14]	2017/Norway	Lancet Neurology	549 vs 551	RCT	Patients eligible for intravenous thrombolysis as bridging therapy before endovascular treatment were included in the study. Older than 80 years. Minor neurological deficits at presentation. Previous history of stroke and concomitant diabetes mellitus were allowed.	Intravenous tenecteplase 0·4 mg/kg (to a maximum of 40 mg) versus alteplase 0·9 mg/kg (to a maximum of 90 mg)

Comparison of Outcome Measures

Clinical outcome measures (NIHSS at baseline and 24 hours, three-month mRS, ICH incidence on follow-up computed tomography (CT), and mortality) were common to all four studies and shown in Table 2. All studies applied different definitions of SICH. Haley et al. terminated prematurely and did not proceed with the original plan to compare three-month functional outcomes for the selected tenecteplase dose. Parsons et al. used the coprimary endpoints of the proportion of reperfused perfusion lesions measured by CT perfusion (CTP) and the extent of NIHSS score improvement at 24 hours. Recanalization status, penumbral salvage, and infarct volume at 24 h were additionally available for two studies.

**Table 2:** Clinical outcome in patients taking tenecteplase MNI: Major neurological improvement mRS: Modified ranking scale SICH: Symptomatic intracerebral hemorrhage

Study	Early MNI No. (%)	mRS at 90 days (0-1) no. (%)	SICH No. (%)	Mortality in 90 days No. (%)
Haley et al. [9]	7 of 31 (22.6% ) in 0.1-mg/kg tenecteplase group, 11 of 31 (35.5%) in 0.25-mg/kg tenecteplase group, 4 of 19 (21.1% ) in 0.4-mg/kg tenecteplase group, and 5 of 31 (16.1%) in alteplase group	14 of 31 (45.2%) in 0.1-mg/kg tenecteplase group, 15 of 31 (48.4%) in 0.25-mg/kg tenecteplase group, 7 of 19 (36.8%) in 0.4-mg/kg tenecteplase group, and 13 of 31 (41.9%) in alteplase group	Present in 3 of 19 (15.8%) in the 0.4-mg/kg group, 2 of 31 (6.5%) in the 0.25-mg/kg tenecteplase group, 0 of 31 in the 0.1-mg/kg tenecteplase group, and 1 of 31 (3.2%) in the alteplase group	2 of 31(6.5% ) in the 0.1-mg/kg group, 7 of 31 (22.6%) in the 0.25-mg/kg group, 3 of 19 (15.8%) in the 0.4-mg/kg group and 8 of 31(25.8%) in the alteplase group
Parsons MW et al. [10]	32 (64%) in tenecteplase group vs 9 (36%) in alteplase group, p = 0.02	27 (54% ) in tenecteplase group vs 10 (40%) in alteplase group, p= 0.25	2 (4%) in tenecteplase group vs 3 (12%) in alteplase group, p= 0.33	4 (8%) in tenecteplase group vs 3 (12%) in alteplase group, p= 0.68
Huang et al. [11]	19/47 (40%) in tenecteplase vs 12/49 (24%) in alteplase, p=0.10	7/47 (15%) in tenecteplase vs 7/49 (15%) in alteplase, p= 0.89	ECASS II definition : 3/52 (6%) in tenecteplase vs 4/51 (8%) in alteplase, p=0.59 SITS-MOST definition : 1/52 (2%) in tenecteplase vs 2/51 (4%) in alteplase, p=0.50	8/47 (17%) in tenecteplase vs 6/49 (12%) in alteplase, p= 0.51
Logallo et al. [14]	229/549 (42%) in tenecteplase vs 214/551 (39%) in alteplase, p=0.97	354/549 (64%) in tenecteplase vs 345/551 (63%) in alteplase, p= 0.52	15/549 (3%) in tenecteplase vs 13/551 (2%) in alteplase, p= 0.70	29/549 (5%) in tenecteplase vs 26/551 (5%) in alteplase, p=0.68

Efficacy and Safety

In the study of Haley et al., the highest proportion of early major neurological improvement was seen in the 0.25-mg/kg tenecteplase group (11 of 31 (35.5%)) and the lowest proportion in the alteplase group (5 of 31 (16.1%)). In terms of good mRS outcome (0-1), the 0.25-mg/kg tenecteplase group had the highest proportion (15 of 31 (48.4%)), and the 0.4-mg/kg tenecteplase group had the lowest (7 of 19 (36.8%)). The highest proportion of SICH was present in the 0.4-mg/kg tenecteplase group (3 of 19 (15.8%)). Mortality at three months was highest for the alteplase group (8 of 31 (25.8%)) and for the 0.25-mg/kg tenecteplase group (7 of 31 (22.6%)) [9].

Parsons et al. evaluated that early major neurological improvement was higher in the tenecteplase group (32 (64%)) vs. the alteplase group (9 (36%)). The study found that 27 (54%) patients in the tenecteplase group and 10 (40%) in the alteplase group achieved the primary outcome of mRS score of 0–1 points at three months. Symptomatic intracerebral hemorrhage was seen in two (4%) in the tenecteplase group and three (12%) in the alteplase group. By three months, four patients (8%) had died in the tenecteplase group compared with three (12%) in the alteplase group [10].

In the study of Huang et al., the percentage of penumbra salvaged did not differ significantly between the alteplase and tenecteplase groups. No significant differences were noted for any secondary endpoints, either for imaging or for clinical outcomes [11]. In this study, neurological and radiological outcomes did not differ between the tenecteplase and alteplase groups. Intracerebral hemorrhage of any kind was seen in eight patients (15%) in the tenecteplase group and 14 (27%) in the alteplase group (OR 0.4 (95% CI 0.2–1.2); p=0.09). Only one patient (2%) in the tenecteplase group had a parenchymal hemorrhage compared to five (10%) in the alteplase group. The incidence of symptomatic intracerebral hemorrhage, with either the SITS-MOST [12] definition or the ECASS II [13] definition, did not differ between treatment groups [11].

Logallo et al. evaluated that 354 (64%) of 549 patients in the tenecteplase group and 345 (63%) of 551 patients in the alteplase group achieved the primary outcome of mRS score 0–1 points at 3 months (OR 1.08, 95% CI 0.84–1.38; p=0.52). There was no difference in major neurological improvement at 24 hours or the ordinal shift analysis at three months [14]. During the first 24–48 hours after thrombolytic treatment, any intracranial hemorrhage occurred in 47 patients (9%) in the tenecteplase group and 50 patients (9%) in the alteplase group (OR 0.94, 95% CI 0.60–1.45; p=0.82), and symptomatic intracranial hemorrhage in 15 (3%) and 13 (2%) patients, respectively (OR 1.16, 95% CI 0.51–2.68; p=0.70). By three months, 29 (5%) of 549 patients had died in the tenecteplase group compared with 26 (5%) of 551 in the alteplase group (OR 1.12, 95% CI 0.63–2.02; p=0.68) [14]. The author had concluded that tenecteplase was not superior to alteplase for the treatment of acute ischemic stroke and had a similar safety profile.

Only one systematic review and meta-analysis have been performed to compare the efficacy and safety of tenecteplase vs. alteplase. The review published in 2016 by Xuya Huang et al. included three randomized, controlled trials from 1994-2015 using different sources. The review included data from 291 participants with a confirmed diagnosis of ischemic stroke. There were no differences between any dose of tenecteplase and alteplase for either the efficacy or safety end points. Tenecteplase 0.25 mg/kg had the greatest odds of achieving early neurological improvement (OR (95%CI) 3.3 (1.5, 7.2), p = 0.093), excellent functional outcome (modified Rankin Scale 0–1) at three months (OR (95%CI) 1.9 (0.8, 4.4), p = 0.28), with reduced odds of intracerebral hemorrhage (OR (95%CI) 0.6 (0.2, 1.8), P=0.43) compared with alteplase. Tenecteplase 0.4 mg/kg showed increased odds of symptomatic intracerebral hemorrhage compared with alteplase (OR (95% CI) 6.2 (0.7, 56.3)) [15].

The present work is, to our knowledge, the only first systematic review of the latest four randomized controlled trials, allowing a direct comparison of the efficacy and safety of TNK and alteplase in patients with ischemic stroke. Despite the relatively small number of available original studies, the number of included patients was large. The aim of this systematic review was to identify if tenecteplase is more efficacious and safer than alteplase.

Based on the studies analyzed, one thing that is found in common is that tenecteplase is not more efficacious nor safer than alteplase in the management of ischemic stroke. One of the key point or issues to start any intervention is the efficacy and safety. We think that there is a possibility of a dose-dependent efficacy and safety of recombinant tissue plasminogen activator (rtPA) [16]. Efforts to select the optimal tenecteplase dose were methodologically rigorous, but premature trial discontinuation limits the reliability of dose selection and likely contributes to the variability of doses being investigated in current and upcoming tenecteplase studies [9]. Early major neurological improvement and functional independence at 90 days were likely to be associated with tenecteplase 0.25 mg/kg dose [9-11]. There might be possible existence of a dose-dependent ICH risk and ICH was likely to be associated with 0.4 mg/kg tenecteplase [9,14].

In a systematic review about safety and efficacy of tenecteplase versus alteplase in acute coronary syndrome (ACS), tenecteplase was associated with a reduced risk of major bleeding when compared to alteplase in ACS without evidence of reduced efficacy [17]. Similarly, a systematic review of thrombolytic therapy for acute pulmonary embolism (PE) reported a lower risk of major bleeding in the patients receiving alteplase when compared to those receiving tenecteplase [18]. The assessment of the safety and efficacy of a new thrombolytic (ASSENT-2) trial reported that tenecteplase and alteplase were equivalent for 30-day mortality in myocardial infarction but the ease of administration of tenecteplase may facilitate more rapid treatment in and out of the hospital [4].

Delays between initial bolus and the initiation of maintenance infusion are common with alteplase and might compromise the effectiveness [19-20]. In the tertiary level hospitals of Nepal, tenecteplase is easily available and the price of tenecteplase ($450) is half the price of alteplase ($1000). In a country where the per capita income is just $691.7 and 25.2% population fall below the poverty line, tenecteplase might be a better option than alteplase for the management of acute ischemic stroke.

## Conclusions

This systematic review infers that tenecteplase is equivalent to alteplase in its efficacy and safety while managing acute ischemic stroke. The use of tenecteplase 0.25 mg/kg is clinically justified and is similar in efficacy and safety to alteplase 0·9 mg/kg. However, the ease of administration (just an intravenous bolus and no maintenance infusion) could offer an advantage to tenecteplase over alteplase. In low-income countries like Nepal, the patient’s compliance with alteplase could decrease in large amounts because of its huge cost and lower availability when compared to tenecteplase. So, tenecteplase can be a better option than alteplase for the management of acute ischemic stroke in the low-income country, Nepal.
